# The complete plastid genome of *Amphicarpaea ferruginea* Bentham (Leguminosae), a grass species with development and utilization prospect

**DOI:** 10.1080/23802359.2022.2089603

**Published:** 2022-07-07

**Authors:** Xiao Yu, Zhao Zhen-Ning, Ping Huai-Lei

**Affiliations:** a School of Landscape Architecture and Horticulture Sciences, Southwest Forestry University, Kunming, China; b School of Forestry, Southwest Forestry University, Kunming, China

**Keywords:** *Amphicarpaea ferruginea*, grass species, plastid genome, phylogenetic analysis

## Abstract

We are reporting the complete plastid genome of *Amphicarpaea ferruginea*, a grass species with development and utilization prospect. The *A. ferruginea* plastome is 152,531 bp long, with two inverted repeat(IR) regions (25,616 bp each) that separate a large single copy (LSC) region (83,364 bp) and a small single copy (SSC) region (17,935 bp). A total of 130 genes were annotated, including 85 protein-coding genes, 8 rRNA genes, and 37 tRNA genes. The phylogenetic tree shows that *Amphicarpaea edgeworthii* is closely related to *Amphicarpaea ferruginea* with strong bootstrap support.

*Amphicarpaea ferruginea* Bentham (1852) is a perennial herbaceous vine belonging to the genus *Amphicarpaea* in Leguminosae, mainly distributed in Assam state in India, central and southern China, Eastern Himalayas, Myanmar, Nepal, and Thailand (Wu 1995; Kumar and Sane 2003). Due to the unique developmental pattern of the *Amphicarpaea* species plant, it has become a model plant for the study of plant developmental biology, which has unique significance of breeding (Shan et al. 2009). The morphological characteristics of the *Amphicarpaea* and *Glycine* plant are very similar, and they also have a very close relationship of each other (Ohashi and Ohashi 2016). Scientists hope to find the trait genes that control the way *Amphicarpaea* species develop, allowing soybeans to produce both above-ground and below-ground results to increase yields (Ohashi and Ohashi 2018). In addition, *Amphicarpaea* species plants are rich in crude protein, calcium and phosphorus, have high forage to value, and are potential high-quality forage resources (Jiang et al. 2013). The seeds contain isoflavones, which have anti-inflammatory, antioxidant, anti-tumor, antibacterial and other effects (Jiang et al. 2007). *A. ferruginea* is one of the leguminous grass species with development and utilization prospects in the central and southern subtropical regions. In this study, we characterized a complete plastid genome of *A. ferruginea* and confirmed the phylogenetic relationship of the genus, to provide genetic information for further research on phytogeography, genetic diversity and evolution.

The fresh leaves of *A. ferruginea* were collected from Nanxi village, Huangshan Town, Yulong County, Yunnan Province, China (coordinates:100°8'59.93″E, 26°46'8.02″N; altitude: 3103 m). The collection of plant materials complies with the wild plant protection regulations of the people's republic of China and obtain the permission of local authorities on forestry and grassland bureau Yunnan province in China. The voucher specimen (SWFU20210776MFY) was deposited at Herbarium of Southwest Forestry University, China (http://bbg.swfu.edu.cn/, Yu Xiao, email:yuxiao0215@gmail.com). Total genome DNA was extracted with the Ezup plant genomic DNA preps Kit (Sangon Biotech, Shanghai, China). A total of 3 G raw data from Illumina Hiseq Platform (Illumina, San Diego, CA) were sequenced. Then the raw data was used to assemble the complete chloroplast genome using the software of GetOrganelle (Jin et al. 2020). Annotated using Geneious Prime (Kearse et al. 2012) with reference to the complete plastid genome sequence of *A. edgeworthii* (NC_057598.1). The complete plastid genome of *A. ferruginea* was submitted to GenBank with accession number ON050971.

The plastome of *A. ferruginea* is a double-stranded circular DNA with the length of 152,531 bp, containing a large single copy (LSC) region of 83,364 bp, a small single copy (SSC) region of 17,935 bp, and a pair of inverted repeats of 25,616 bp. The base compositions of the cp genome were uneven (32.26% A, 17.60% C, 17.85% G, and 32.29% T). The GC and AT content of the whole plastome is 35.44% and 64.56%. GC content in IR region (41.88%) was higher than that in LSC region (32.91%) and SSC region (39.11%). The plastid genomes were annotated with 130 genes, including 85 protein-coding genes, 37 tRNA genes, and 8 rRNA genes. Eighteen genes replicate in the IR region and repeat inversely with each other, including seven protein-coding genes (*rpl2*, *rpl23*, *ycf2*, *ndhB*, *rps7*, *rps12*, *ycf1*), seven tRNA genes (*trnN-GUU*, *trnI-CAU*, *trnL-CAA*, *trnA-UGC*, *trnV-GAC*, *trnI-GAU*, *trnR-ACG*), and four rRNA genes (*rrn4.5*, *rrn5*, *rrn16*, *rrn23*). A total of 73 SSRs were discovered by the online software MISA-web (Beier et al. 2017). Among them, the numbers of mono-, di-, tri-, tetra-, and pentanucleotides SSRs are 41, 28, 3, 1, and 0, respectively.

A phylogenetic tree was reconstructed to confirm the phylogenetic location of *A. ferruginea.* Two species of *Cycas debaoensis* and *Cycas szechuanensis* were used as out-groups. All of these 24 complete cp sequences were aligned by the MAFFT version 7 software (Katoh and Standley 2013). A maximum likelihood method for phylogenetic analysis was performed base on GTR + I + G model in the RAxML version 8 program with 1000 bootstrap replicates (Darriba et al. 2012; Stamatakis 2014). Phylogenetic analysis results strongly supported that *A. ferruginea* was sister related to the *A. edgeworthii* (Figure 1). The complete chloroplast genome sequence of *A. ferruginea* will provide useful information for further study on genetic diversity and conservation of *Amphicarpaea* species.

**Figure 1.**
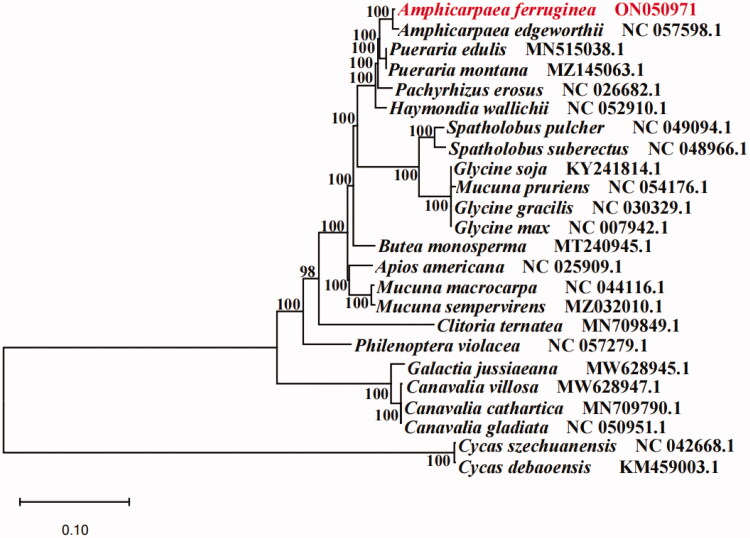
The maximum likelihood (ML) phylogenetic tree of *Amphicarpaea ferruginea* and 21 relative species were reconstructed by RAxML based on complete chloroplast genome sequences. The bootstrap support value is labeled for each node.

## Author contributions

X. Y. conceived the study, collected the molecular materials, and drafted the manuscript; Z. N. Z. and H. L. P. analyzed the experimental data. All authors provided comments and final approval.

## Data Availability

The genome sequence data that support the findings of this study are openly available in GenBank of NCBI at https://www.ncbi.nlm.nih.gov under the accession no.ON050971. The associated BioProject, SRA, and Bio-Sample numbers are PRJNA820613, SRR18500621, and SAMN27019003, respectively.
